# Articular Cartilage Gene Expression after Coxofemoral Joint Luxation in the Dog

**DOI:** 10.1155/2013/936317

**Published:** 2013-09-30

**Authors:** Korakot Nganvongpanit, Waranee Pradit, Siriwadee Chomdej

**Affiliations:** ^1^Animal Bone and Joint Research Laboratory, Department of Veterinary Biosciences and Public Health, Faculty of Veterinary Medicine, Chiang Mai University, Chiang Mai 50100, Thailand; ^2^Department of Biology, Faculty of Science, Chiang Mai University, Chiang Mai 50200, Thailand

## Abstract

This study examined the relationship between days of hip luxation and the expression of various mRNA. Twenty-six articular cartilages were used in the experiment: 3 samples were from normal dogs and 23 samples were collected from the femoral heads of hips that had been luxated for different lengths of time. Ten mRNA, including nonapoptotic genes *(AGG, COL2A1, MMP-3, HAS-1, HAS-2, and TIMP-1)* and apoptotic genes *(BAX, BCL-2, CAS-3, and CAS-9)*, were studied for their expression using real-time PCR. We found very high correlation between expression level and luxation days (*r* ^2^ > 0.9) in *COL2A1, MMP-3, HAS-1, HAS-2, TIMP-1, BAX, and CAS-9*, while the others *(AGG, BCL-2, and CAS-3)* also showed high correlation (*r* ^2^ = 7–9). And we found a significant difference (*P* < 0.05) in the expression of transcripts depending on the number of luxation days. In conclusion, a delay in joint reduction may increase the chances of development of osteoarthritis.

## 1. Introduction

Coxofemoral luxation (hip luxation) is relative common orthopedic problem in dogs. The major causes of this problem are accidents (vehicle accidents ranged from 59 to 83%) and falls from height [1]. In our data recorded since 2006, 80% were caused by vehicle accidents, 10% caused by falling from height, 7% caused by dog bite, and 3% caused by their leg became caught in the wires of a cage (data not yet published). It is well known that ligament rupture, joint instability, and articular cartilage injury can cause osteoarthritis (OA). Articular cartilage and surrounding soft tissues (i.e., tendon, ligament, or joint capsule) are injured when the hip joint is luxated, and during the reduction procedure as well as is well known, the goals of treatment for luxation of the hip are to reduce the dislocation with as little damage to the articular surface as possible and to stabilize the joint sufficiently to allow soft tissue healing, with the expectation of normal clinical function. The first choice of treatment of simple joint luxation is reduction (closed or open), but in severe cases involving ligament rupture, surgery is required to repair the ligament and joint capsule [2]. 

To predict the chances of a joint developing OA, several criteria need to be considered. However, we believe that the period of luxation before reduction is another important criterion because it has a direct effect on articular cartilage metabolism. During joint luxation, the articular cartilage is unable to receive nutrients from, or release waste products into, the synovial fluid. This is the cause of the OA mechanism in joints [3, 4]. This study aims to quantify the expression of certain genes which are related to the OA mechanism. Furthermore, the results of this study could provide useful information for predicting the chances of developing OA after joint reduction.

## 2. Methods

### 2.1. Sample Collection

Twenty-six canine articular cartilages were used in this study. Three cartilages were harvested from normal cadavers, and 23 cartilages were harvested from the femoral neck during femoral head osteotomy procedure in dogs undergoing treatment at an animal hospital for coxofemoral luxation. All femoral heads used in this study were normal. Dogs with prior coxofemoral luxation, septic arthritis, osteoarthritis, or hip dysplasia were excluded from this study. The number of days of luxation was recorded for each sample. The experimental protocol was approved by the Faculty of Veterinary Medicine and the Ethics Committee, Chiang Mai University, Thailand.

### 2.2. RNA Isolation and Synthesis of cDNA

RNA isolation and purification of each sample, including the DNA removal step, were performed using an RNeasy Mini Kit protocol (Qiagen, Hilden, Germany), according to the manufacturer's guidelines. RNA was eluted in 40 *μ*L of RNase-free water (Qiagen). Reverse transcription was performed using 10 *μ*L RNA with oligo (dT) 12–18 primer and SuperScript II reverse transcriptase (Invitrogen, Karlsruhe, Germany). First, mRNA and oligo (dT) primer were mixed, heated to 70°C for 3 min, and placed on ice until the addition of the remaining reaction components. The reaction was then incubated at 42°C for 90 min and terminated by heat inactivation at 70°C for 15 min.

### 2.3. Quantitative Real-Time PCR

Quantification of ten transcripts—aggrecan (*AGG*); type II collagen, alpha-1 chain (*COL2A1*); matrix metalloproteinase-3 (*MMP-3*); hyaluronan synthase-1 (*HAS-1*); hyaluronan synthase-2 (*HAS-2*); tissue inhibitor of metalloproteinase-1 (*TIMP-1*); Bcl-2-associated X protein (*BAX*); B-cell lymphoma-2 (*BCL-2*); cysteine aspartate-specific protease-3 (*CAS-3*); and cysteine aspartate-specific protease-9 (*CAS-9*) (Shanghai BlueGene Biotech, Shanghai, China)—was performed for all samples. An ABI Prism 7000 sequence detection system (Applied Biosystems, Foster City, CA, USA) was used for quantitative analysis using SYBR Green JumpStart Taq ReadyMix (Sigma-Aldrich, St. Louis, MO, USA), incorporating dsDNA-specific fluorescent detection dye. Quantitative analyses of all transcriptions were performed in comparison with glyceraldehyde-3-phosphate dehydrogenase (*GAPDH)* as an endogenous control and were run in separate wells [5]. PCR was performed using 2 *μ*L of each sample of cDNA and specific amplification primers. The primer sequences were designed for PCR amplification according to the human cDNA sequence (Table 1) using Primer Express software v. 2.0 (Applied Biosystems). Standard curves were generated for both target and endogenous control genes using serial dilutions of plasmid DNA (10^1^–10^8^ molecules). The PCRs were performed in 20 *μ*L reaction volume containing 10.2 *μ*L SYBR Green PCR Master Mix, optimal levels of forward and reverse primers, and 2 *μ*L of embryonic cDNA. During each PCR, reaction samples from the same cDNA source were run in duplicate to control the reproducibility of the results. A universal thermal cycling parameter (an initial denaturation step at 95°C for 10 min and 45 cycles of denaturation at 95°C for 15 s and 60°C for 60 s) was used to quantify each gene of interest. After the end of the last cycle, a dissociation curve was generated by starting the fluorescence acquisition at 60°C and taking measurements at 7 s intervals until the temperature reached 95°C. Final quantitative analysis was done using the relative standard curve method, as in previous reports [5, 6]. Results were reported as the relative expression level compared to the calibrator cDNA after normalization of the transcript amount to the endogenous control.

### 2.4. Statistical Analysis

The mRNA expression analysis for studied genes was based on the relative standard curve method. The relative expression data were analysed using the SAS version 8.0 software package. Pearson's correlation was used to examine the relationship between luxation days and gene expression levels. A statistical difference of *P* ≤ 0.05 was considered to be significant.

## 3. Results

### 3.1. Animal Data

Twelve male and 14 female dogs included Bangkaew (*n* = 2), Pomeranian (*n* = 5), Shih-Tzu (*n* = 6), Cocker Spaniel (*n* = 2), Pug (*n* = 2), Chihuahua (*n* = 3), Bulldog (*n* = 1), Poodle (*n* = 3), Thai Ridgeback (*n* = 1), and French Bulldog (*n* = 1). The body weight range was 1.4–20.5 kg (mean 10.05 ± 6.49 kg) and the age range was 15–60 months (mean 40.35 ± 11.68 months).

In this study, of the 23 dogs with luxation, 21 cases resulted from accidents, while 2 cases were of unknown cause. The 21 accident cases included 10 dogs that were injured in vehicle accidents, 5 dogs that were bitten by larger dogs, 2 dogs that fell from height, and 4 dogs when their leg became caught in the cage. 

Periods of luxation included 1 day (*n* = 1), 2 days (*n* = 2), 4 days (*n* = 1), 5 days (*n* = 3), 6 days (*n* = 1), 7 days (*n* = 2), 8 days (*n* = 1), 10 days (*n* = 2), 11 days (*n* = 1), 12 days (*n* = 2), 14 days (*n* = 2), and 18, 19, 21, 23, and 30 days (*n* = 1 per day). 

### 3.2. Relationship between Gene Expression and Days of Luxation

**Figure 1:**
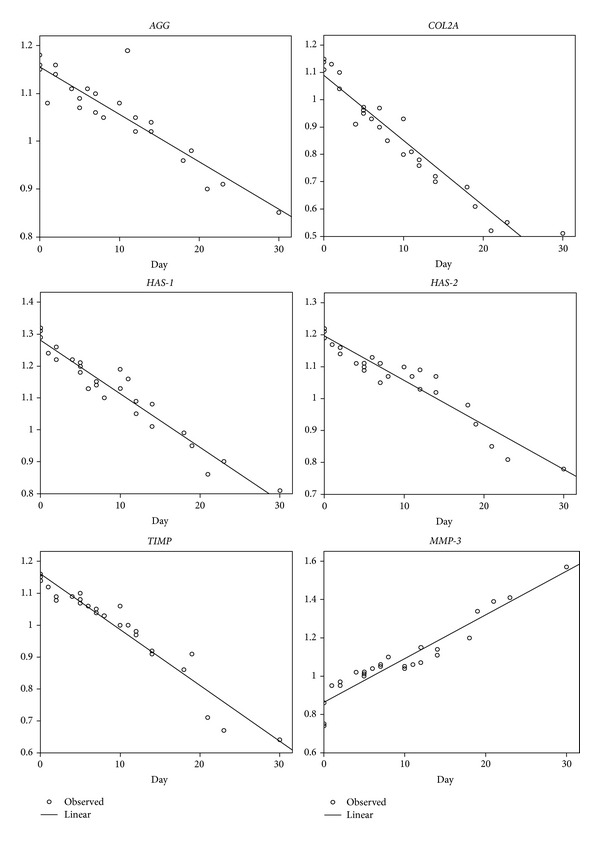
The relationship between relative expression of nonapoptotic genes and days of luxation (*AGG*, aggrecan; *COL2A*, type II collagen (alpha-1 chain); *HAS-1*, hyaluronan synthase-1; *HAS-2*, hyaluronan synthase-2; *TIMP*, tissue inhibitor of metalloproteinase; *MMP-3*, matrix metalloproteinase-3).

**Figure 2:**
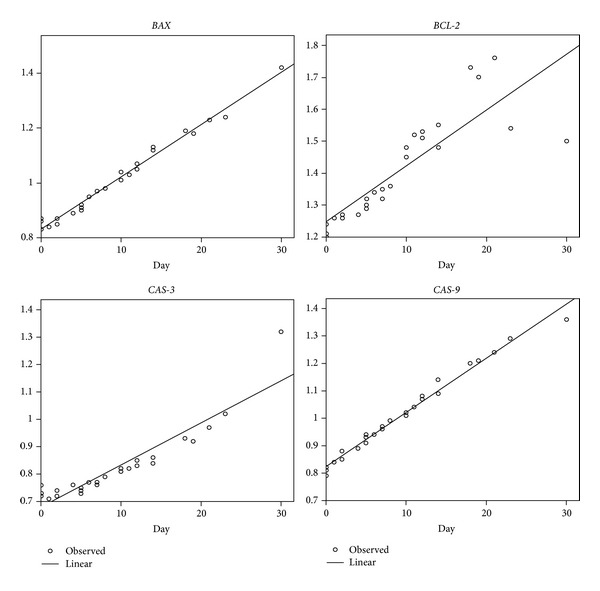
The relationship between relative expression of apoptotic genes and days of luxation (*BAX*, Bcl-2-associated X protein; *BCL-2*, B-cell lymphoma-2; *CAS-3* cysteine aspartate-specific protease-3; *CAS-9*, cysteine aspartate-specific protease-9).

The relative expressions of *AGG, COL2A1, HAS-1, HAS-2, *and* TIMP-1* were downregulated, while relative expressions of *BCL-2, BAX, CAS-3, CAS-9, *and* MMP-3* were upregulated when the number of days of joint luxation was increased (Figures 1 and 2). 

The relative expression levels of genes were used to find the correlation between level of expression and days of luxation. The linear regression equation *R*, *R* ^2^, and the significance level of each gene are shown in Table 2. A very high correlation (>0.9) was found in *COL2A1, MMP-3, HAS-1, HAS-2, TIMP-1, BAX, *and* CAS-9*, while the others (*AGG, BCL-2, *and* CAS-3*) also showed high correlation. Moreover, expression levels of all genes were significantly related (*P* < 0.001) to days of luxation. 

## 4. Discussion

This study is the first report demonstrating a correlation between the expression levels of candidate genes and the number of days of coxofemoral luxation. Genes responding to anabolism, including *AGG, COL2A1, HAS-1, HAS-2, *and* TIMP-1*, were downregulated, while *MMP-3* was upregulated. Moreover, four apoptosis transcripts, *BAX, BCL-2, CAS-3,* and *CAS-9*, were upregulated with increased days of luxation. Moreover, we found that the expression levels of all genes showed a significant change depending on the number of days of luxation.

The study design had some limitations. First, this study was clinical research; samples were collected from dogs that had come to the hospital for surgery to remove the femoral head. Hence the samples were randomly varied in terms of the number of days of luxation. Also, we could not control the number of samples collected on a given day (some days more than two, some days only one). Second, we were unable to obtain articular cartilage from the acetabulum to study gene expression and to compare with gene expression from the femoral head. Third, the sample was not large enough to study apoptosis by TUNEL assay because all cartilage was used to extract mRNA. 

OA is characterized by a loss of cartilage matrix and chondrocytes as well. The actions of proteolytic enzymes (MMPs) degrade both proteoglycans and collagen. Moreover, hypertrophic chondrocyte phenotype in OA increased production of MMPs, collagen type X, and alkaline phosphatase [7]. This study chose genes to represent different mechanisms, including the anabolic pathway (*AGG, COL2A1, HAS-1, HAS-2,* and *TIMP-1*), catabolic pathway (*MMP-3*) and apoptosis pathway (*BAX, BCL-2, CAS-3,* and *CAS-9*). Expression level changes in those genes can be used as a predictor of OA.

The *AGG* gene was found to exhibit a unique feature, in that the core proteins had the capacity to interact with glycosaminoglycans (GAG) and hyaluronic acid (HA) [8]. We found that the expression of *AGG* was significantly decreased during the luxation period and was highly correlated with days of luxation (*r* ^2^ = 0.808). During the OA process, aggrecans are cleaved at specific Glu-Xaa peptide bonds by aggrecanases such as ADAMTS-4 and ADAMTS-5, resulting in a breakdown of HA and link proteins and a decrease in stable ternary complexes in the extracellular matrix [9]. However, we did not quantify the level of ADAMTS-4 or ADAMTS-5 to confirm the mechanism, which should increase during the luxation period. To represent this process, this study used only *MMP-3*, which showed an increase during the period of luxation. 

*HAS-1 *and* HAS-2* genes are capable of directing the synthesis of HA. *HAS-2* has been found to be most abundant in articular chondrocytes; synovial cells show an opposite trend, with *HAS-1* always being more abundant than *HAS-2* [10]. Our study found that levels of *HAS-1 *and* HAS-2* were significantly correlated with days of luxation (*r* ^2^ = 0.936 and 0.908, resp.). 

*MMP-3* acts to degrade the extracellular matrix (ECM): proteoglycans, gelatin, laminin, fibronectin, and collagen (types III, IV, and IX). Moreover, *MMP-3* can stimulate other enzymes in the MMP group, such as *MMP-1, MMP-7, MMP-8, MMP-9,* and *MMP-13*. This stimulation increases biochemical substance degradation, including degradation of type II collagen, the most important type of collagen in the ECM [11]. This study found that expression of *MMP-3* was significantly higher during the period of luxation; moreover, this expression was highly correlated with days of luxation (*r* ^2^ = 0.907). It is well known that the activity of MMPs is controlled by TIMPs, and an imbalance between MMPs and TIMPs is of great importance in the progression of OA [12]. Our study found that the *TIMP-1* gene level was significantly different during the luxation period and was highly correlated with days of luxation (*r* ^2^ = 0.930). 

Programmed cell death (PCD) is a precisely coordinated event dependent upon the actions and interactions of a number of gene products that either suppress or activate the process of cellular self-destruction. Currently there are more than 100 different genes whose expressions affect cell survival [13]. The major mechanism of chondrocyte apoptosis includes the involvement of *Fas*, *TNF*, TNF-related apoptosis-inducing ligand-(*TRAIL-*)* R1*, *TRAIL-R2*, and nitrous oxide (*NO*) exposure [14]. These induce the apoptosis pathway throughout many genes, including the caspase family (*Caspase-2, -3, -6, -7, -8, -9, *and* -10*), and proapoptotic (*BAX, BAD*) and antiapoptotic members (*BCL-2*) [15].

Caspase, a family of cysteine proteases, mediates proteolytic cleavage of a larger number of proteins, leading to the apoptotic morphology observed in cells [16]. Caspases are central players in apoptosis because they catalyze many steps in the death pathway by cleavage at specific sites containing aspartic acid [17]. At least two general classes of apoptotic caspases exist. Initiator caspases (*Caspase-2*, -*8*, -*9*, and -*10*) are present in complexes with other regulatory proteins and are activated by facilitated autocatalysis in response to apoptotic signals. The other class, effector caspases (*Caspase-3*, -*6*, and -*7*), are activated in a cascade through cleavage by initiator caspases [18]. Effector caspases then cleave a number of specific substrates, leading to destruction of cell-to-cell interactions and nuclear structure; reorganization of the cytoskeleton; inhibition of DNA synthesis, repair, and splicing; degradation of DNA; and disintegration of the entire cell contents into apoptosis [18].

The *BCL-2* family, which is involved in the regulation of caspase activity, is subdivided into proapoptotic (*BAX, BAD*) and antiapoptotic members (*BCL-2*), which share one or more similar regions [19, 20]. The balance between the life and death of cells may depend on the relative levels of pro- and antiapoptotic members. The mechanism of action of the *BCL-2* family almost certainly involves changes in mitochondrial permeability, either by mitochondrial swelling and physical disruption or by pore formation [19]. Apoptotic mitochondrial changes are mainly prevented by antiapoptotic proteins such as Bcl-2, located in the outer mitochondrial membrane as well as the nuclear envelope and endoplasmic reticulum. However, during apoptosis, proapoptotic members are activated and translocated from the cytosol to the mitochondria [21]. In this way, the Bcl-2 family of proteins is believed to regulate the release and activation of proapoptotic factors from mitochondria, such as cytochrome-c and apoptosis-inducing factors, which lead to the activation of caspases and other downstream execution proteins and, ultimately, cell death. This study found that expression levels of *BAX *and* BCL-2* were highly correlated with days of luxation (*r* ^2^ = 0.986 and 0.715, resp.).

In conclusion, we found that the number of days of luxation is related to articular cartilage gene expression. As the number of days of luxation increased, genes controlling cartilage matrices were significantly decreased, while genes controlling enzymes and apoptosis pathways were significantly increased. These findings suggest that a longer period of luxation of the hip joint increases the chances of developing OA. 

## Figures and Tables

**Table 1:** Sequences of sense and antisense primers used for amplification in real-time PCR.

Gene	Accession number	Primer sequence (5′→ 3′)	Temp. °C	Amplicon size (bp)
*AGG *	U65989_2	Rw: ACTGCTCCAGGCGTGTGATG Fw: GACCATGTCGTGCAGGTGAC	58	405
*BAX *	NM_001003011	Rw: TGCTGGCAAAGTAGAAGAGGGCAA Fw: TTCCGAGTGGCAGCTGAGATGTTT	60	79
*BCL-2 *	NM_001002949	Rw: GTGCTTTGCATTCTTGGATGAGGG Fw: CATGCCAAGAGGGAAACACCAGAA	62	76
*CAS-3 *	NM_001003042	Rw: TTCTGACAGGCCATGTCATCCTCA Fw: TTCATTATTCAGGCCTGCCGAGG	62	83
*CAS-9 *	NM_001031633	Rw: TTGTTGATGATGAGGCAGTAGCCG Fw: TCAGTGACGTCTGTGTTCAGGAGA	61	97
*COL2A1 *	AF023169	Rw: TGCTTTCCAGTTGGGCCAGC Fw: GAGCTCCTGGTGCATCTGGA	58	233
*MMP-3 *	AY183143_1	Rw: CAGAGCTTTCTCAATGGCAG Fw: CTCACCCAGCAATACCTAGA	55	297
*GAPDH *	DQ403060	Rw: CGAAGTGGTCATGGATGACT Fw: AGTATGATTCTACCCACGGC	55	362
*HAS-1 *	XM_849398	Rw: GCATAGAAGAGCCGCAACAC Fw: CAGACACGCTGGTCCAAATC	55	149
*HAS-2 *	XM_539153	Rw: GACTCATCCGTCTCACCAG Fw: GGTCATAGATGGGAACTCG	51	135
*TIMP-1 *	AF077817_1	Rw: TGTCACTCTGCAGTTTGCAG Fw: GATGTTCAAGGGTTTCAGCG	55	294

**Table 2:** Linear regression equation *R*, *R* ^2^, and significance level (Sig.) of candidate genes.

Gene	Equation	*R*	*R* ^2^	Sig.
*AGG *	*y* = − 0.016*x* + 1.188	0.899	0.808	0.000
*COL2A1 *	*y* = − 0.039*x* + 1.172	0.963	0.928	0.000
*MMP-3 *	*y* = 0.036*x* + 0.800	0.953	0.907	0.000
*HAS-1 *	*y* = − 0.027*x* + 1.334	0.967	0.936	0.000
*HAS-2 *	*y* = − 0.023*x* + 1.245	0.953	0.908	0.000
*TIMP-1 *	*y* = − 0.029*x* + 1.226	0.965	0.930	0.000
*BAX *	*y* = 0.031*x* + 0.760	0.993	0.986	0.000
*BCL-2 *	*y* = 0.029*x* + 1.180	0.846	0.715	0.000
*CAS-3 *	*y* = 0.025*x* + 0.625	0.928	0.861	0.000
*CAS-9 *	*y* = 0.032*x* + 0.751	0.993	0.985	0.000

*AGG*: aggrecan; *COL2A1*: type II collagen, alpha-1 chain; *MMP-3*: matrix metalloproteinase-3; *HAS-1*: hyaluronan synthase-1; *HAS-2*: hyaluronan synthase-2; *TIMP-1*: tissue inhibitor of metalloproteinase-1; *BAX*: Bcl-2-associated X protein; *BCL-2*: B-cell lymphoma-2; *CAS-3*: cysteine aspartate-specific protease-3; *CAS-9*: cysteine aspartate-specific protease-9.
